# The SNAPSHOT study protocol: SNAcking, Physical activity, Self-regulation, and Heart rate Over Time

**DOI:** 10.1186/1471-2458-14-1006

**Published:** 2014-09-26

**Authors:** David McMinn, Julia L Allan

**Affiliations:** Rowett Institute of Nutrition and Health, School of Medicine and Dentistry, University of Aberdeen, Health Sciences Building, Foresterhill Campus, Ashgrove Road, Aberdeen, AB25 2ZD Scotland; School of Medicine and Dentistry, University of Aberdeen, Health Sciences Building, Foresterhill Campus, Ashgrove Road, Aberdeen, AB25 2ZD Scotland

**Keywords:** Snack, Food choice, Executive function, Self regulation, Intentions, Physical activity, Ecological momentary assessment, Accelerometer, GPS, PRO-Diary, Actiheart

## Abstract

**Background:**

The cognitive processes responsible for effortful behavioural regulation are known as the executive functions, and are implicated in several factors associated with behaviour control, including focussing on tasks, resisting temptations, planning future actions, and inhibiting prepotent responses. Similar to muscles, the executive functions become fatigued following intensive use (e.g. stressful situations, when tired or busy, and when regulating behaviour such as quitting smoking). Therefore, an individual may be more susceptible to engaging in unhealthy behaviours when their executive functions are depleted. In the present study we investigate associations between the executive functions, snack food consumption, and sedentary behaviour in real time. We hypothesise that individuals may be more susceptible to unhealthy snacking and sedentary behaviours during periods when their executive functions are depleted. We test this hypothesis using real-time objective within-person measurements.

**Methods/Design:**

A sample of approximately 50 Scottish adults from varied socio-economic, working, and cultural backgrounds will participate in the three phases of the SNAcking, Physical activity, Self-regulation, and Heart rate Over Time (SNAPSHOT) study. Phase one will require participants to complete home-based questionnaires concerned with diet, eating behaviour, and physical activity (≈1.5 hours to complete). Phase two will constitute a 2-3 hour psychological laboratory testing session during which trait-level executive function, general intelligence, and diet and physical activity intentions, past behaviour, and automaticity will be measured. The final phase will involve a 7-day ambulatory protocol during which objective repeated assessments of executive function, snacking behaviour, physical activity, mood, heart rate, perceived energy level, current context and location will be measured during participants’ daily routines. Multi-level regression analysis, accounting for observations nested within participants, will be used to investigate associations between fluctuations in the executive functions and health behaviours.

**Discussion:**

Data from the SNAPSHOT study will provide ecologically valid information to help better understand the temporal associations between self-regulatory resources (executive functions) and deleterious health behaviours such as snacking and sedentary behaviour. If we can identify particular periods of the day or locations where self-regulatory resources become depleted and produce suboptimal health behaviour, then interventions can be designed and targeted accordingly.

## Background

In Western developed society, we live in an obesogenic environment, where energy dense foods are readily available and there is a proliferation of labour saving devices and sedentary occupations [1]. This means that individuals who intend to improve or maintain their health must continually choose between engaging in behaviours that are effort free and immediately rewarding (e.g. going for a drive-through meal on the way home from work), and those that are ultimately healthy but require effortful behavioural regulation (e.g. walking or cycling home from work before preparing a freshly-cooked meal).

The cognitive processes responsible for effortful behavioural regulation are known as the executive functions [2]. These are multiple related but distinct cognitive processes involved in the planning, initiation, sequencing, and monitoring of complex goal-directed behaviour [3]. Deficits in these executive functions are associated with elevated body mass index and obesity in children and adults [4–7], increased consumption of fatty foods [8, 9], a reduced ability to adhere to stated dietary intentions [10, 11], and a reduced ability to resist indulging in energy-dense foods [12].

Most research linking executive function to dietary behaviour has focused on stable, trait-level differences in executive function between people. However, executive processing is resource intensive, and as a consequence, is vulnerable to transient within-person fluctuations in efficiency. In one sense, the executive functions operate like a muscle: expending regulatory energy depletes available cognitive resources and leaves individuals less able to exert further regulatory control until resources have been replenished [13, 14]. This has been demonstrated experimentally in a laboratory setting, where it has been shown that participants have reduced capacity to regulate consumption of unhealthy foods following tasks designed to deplete self-control resource [15, 16]. Situations under which executive resources may become depleted include times of stress, tiredness, when extremely busy (trying to do multiple things at once), and when trying to regulate behaviours (e.g. when quitting smoking, trying not to get angry/upset etc.). Multiple factors that replenish resources and improve subsequent attempts at behavioural regulation have been identified, most notably rest/sleep [17] and increased blood glucose through consumption of high glucose foods [18].

This resource-dependent variability in the capacity to regulate behavior has three interesting implications for dietary research:

1. Individuals may be more likely to succumb to dietary temptation at moments when their regulatory resources are temporarily depleted. This has been experimentally demonstrated in the laboratory setting [16].
2. These regulatory failures may be more likely to occur in individuals with low trait levels of executive function (as these individuals would be expected to have lower initial levels of cognitive resource). This has also been demonstrated in the laboratory setting [10].
3. When resources are depleted, individuals may be more likely to consume high energy foods and reduce energy expenditure in an attempt to conserve/replenish their dwindling cognitive reserves. This hypothesis, to our knowledge, remains untested.

In each case, the net result is the same; temporary reductions in executive functioning may lead to increased energy intake and decreased energy expenditure. Intriguingly, this may produce a paradoxical situation for individuals trying to eat less/exercise more, as their actions may serve to reduce the very resources they need to continue to regulate their behaviour, making healthy eating and physical activity more difficult, but even more important.

### Aims

The SNAPSHOT study aims to examine the relationship between dietary intake (specifically snack food consumption), energy expenditure, and executive function in real time and outwith the laboratory using Ecological Momentary Assessment (EMA). If, as hypothesised, temporary depletion of executive resources renders people less able to effortfully regulate their behaviour, and encourages increases in energy intake, we would expect to find that episodes of energy dense snack consumption and sedentary behaviour are immediately preceded by drops in executive efficiency. In order to understand the determinants of any such drops in executive efficiency, variation in environmental, social and contextual factors will be recorded. As such, we will be able to identify factors which, if altered, may help to provide an environment that is supportive rather than disruptive to an individual’s attempts to effortfully regulate their behaviour.

SNAPSHOT will assess snack food consumption in particular because, although snacks are theoretically supplementary to the main diet, they represent a major source of caloric intake [19]. Snack foods are typically highly palatable and because they are usually energy-dense (i.e. they contain comparatively more calories per portion size than other foods) they are easy to over consume, leading to a higher caloric intake and ultimately weight gain [20, 21]. Regarding the physical activity component of the present study, it has been shown that sedentary behaviour is particularly detrimental to health [22], because not only is it associated with the absence of physical activity, it is also frequently associated with snacking; particularly so in the case of television viewing [23–25].

Findings from the SNAPSHOT study will help to address the following specific research questions:

- Do individuals with low trait levels of executive function engage in more high-calorie snacking and sedentary behavior than others?
- Are episodes of high calorie snacking and sedentary behaviour immediately preceded by state reductions in executive function efficiency?
- Is any such effect moderated by higher levels of trait executive function (i.e. do higher initial cognitive reserves protect against depletion)?
- Are reductions in executive function efficiency associated with particular contextual/environmental factors?
- Are episodes of high calorie snacking and sedentary behavior associated with particular contextual/environmental factors independently of executive functioning?
- Is energy dense snacking related to reductions in physical activity in real time (i.e. do individuals appear to simultaneously increase intake and decrease expenditure)?

## Methods/Design

### Ethical approval

Ethical approval for all procedures was granted by the College of Life Science and Medicine Ethical Review Board at the University of Aberdeen (CERB/2012/8/761), and all procedures adhere to the Declaration of Helsinki. All participants will provide written informed consent prior to study participation.

### Target population

We aim to recruit adults from a wide variety of backgrounds in terms of occupation, social status, age, and weight. Potential participants will be excluded if they 1) are taking medication that may alter natural heart rhythms (as heart rate measures will be taken), 2) are not fluent in English, 3) are younger than 18 years, 4) have auditory problems that may prevent them hearing a reminder alarm used with one of the measurement instruments, and 5) have motor problems that may prevent them making speeded button presses during some of the psychological tests.

### Recruitment strategy

Recruitment started in February 2013 and will continue simultaneously with data collection through 2014 until a sample of around 50 participants has been achieved. Participants will be recruited using various methods including 1) study posters and flyers around University of Aberdeen buildings and local businesses, 2) distribution of a press-release to local and national media giving details of the study, and 3) creation of a study website (http://www.abdn.ac.uk/snapshot/) in order to attract potential participants and to provide existing participants with relevant study information. Recruitment materials will contain study team contact details so that interested individuals can request a study information sheet providing additional details on the study protocol and inclusion and exclusion criteria.

### Protocol

The SNAPSHOT study will consist of three phases. The first of these is a home-based phase where participants will be sent three questionnaires about diet and physical activity by post. These questionnaires will take approximately 1.5 hours to complete, and will be returned to the research team using a pre-paid envelope. The second phase will involve a 2-3 hour laboratory visit during which participants complete several written and computerised psychological tests, primarily related to the executive functions. Participants will be invited to take part in phase-2 as soon as is practicably possible following receipt of their completed phase-1 questionnaires, preferably within one or two weeks. Phase three will involve a 7-day real-time measurement period, during which participants will wear an accelerometer, GPS tracker, heart rate monitor, and electronic diary during waking hours in order to establish levels of snack consumption, fruit and vegetable consumption (as a comparator to unhealthy snacking), executive function, mood, physical activity, sedentary behaviour, environmental location, and heart rate. Phase three will commence immediately on completion of the phase two laboratory testing session (i.e. participants will leave the laboratory wearing the various measurement devices), regardless of the day of the week and will run for 7 days. A member of the research team will arrange to collect the measurement equipment from the participant at the end of the 7 days of monitoring, and participants will be offered the opportunity to receive a summary of data from each of the measurement devices and an overview of the aims of the project. A nominal award of £20 vouchers for a well known food and clothes retailer will be provided to participants on completion of the study. This is in recognition of the time and effort required from participants.

### Measures

Multiple known determinants of diet and physical activity (e.g. intentions, eating style, past behaviour, etc) will be assessed in addition to the core executive function measures so that (a) the target behaviours of the sample can be adequately described, (b) known predictors can be controlled for in data analyses, and (c) it is possible to test whether executive function moderates the effect of known predictors (e.g. as has been shown in the relationship between dietary intentions and behaviour; Allan et al. 2011 [10]).

### Home-based phase

#### Eating style

Eating style will be assessed using the 33 item Dutch Eating Behaviour Questionnaire [26]. The DEBQ comprises three subscales measuring; 1) emotional eating, defined as the tendency to eat in response to emotional states such as anxiety or anger, 2) external eating, defined as the tendency to eat in response to external food cues such as the sight or smell of food, and 3) restrained eating, defined as the tendency to overeat following a period of dietary restraint. Responses are made using a 5-point Likert scale. The reliability and validity of the DEBQ has been established [27], and recent research has linked high scores on the external eating subscale to the propensity to eat energy dense snacks during periods of sedentary behaviour [28]. The DEBQ takes 3-4 minutes to complete.

#### Typical dietary intake

Typical dietary intake will be measured using the Scottish Collaborative Group Food Frequency Questionnaire version 6.6. The SCG-FFQ consists of 170 foods and drinks grouped into 19 categories, plus an additional three sections on consumption of foods and drinks not listed in the main questionnaire, dietary supplements, and additional information. Common portion sizes for each food/drink are given (e.g. Bread: 1 medium slice) and participants indicate how many portions of each food they eat a day and how often this consumption occurs over the course of a week. All questions are asked with reference to typical diet over the preceding 2-3 months. Photographs illustrating standard portion sizes are given on the front page of the questionnaire. The resultant data will be used to obtain estimates of each participant’s total caloric intake and consumption of important food groups. The SCG-FFQ has been validated in both young and old adults against 4-day weighed intakes [29, 30] and takes 20-30 minutes to complete.

#### Self-reported physical activity

Self-reported physical activity levels will be assessed using the International Physical Activity Questionnaire (IPAQ). The IPAQ is a 27-item questionnaire used to generate data on physical activity over the last 7 days. Activity data are collected for the following domains: work, house and garden work, travel from place to place, and spare time (recreation, exercise, or sport). For each domain, participants are questioned about the number of days (frequency) and hours and minutes per day (volume) that they spend at different activity intensities (i.e. vigorous and moderate). Additionally, the final two items ask about hours and minutes spent per day sitting on weekdays and weekend days (i.e. sedentary behaviour). The IPAQ has acceptable validity when assessing levels and patterns of activity in healthy adults and correlates well with objective accelerometer-based measures of total activity [31]. The IPAQ takes approximately 5 minutes to complete.

### Laboratory phase

Participants will be invited to attend the laboratory phase of the study in a university campus building for a weekday morning or afternoon slot. If the proposed time does not suit the participant, due to work commitments for example, an evening or weekend slot will be offered.

#### Demographics

Participants will be required to attend the laboratory session in a non-fasted state, wearing regular comfortable clothing. At the beginning of the laboratory session participants’ age, gender, years spent in formal education, highest educational qualification, household income, occupation, occupation physical activity level (sedentary, moderately active, or active), and postcode (zip-code) will be recorded. Height will be measured using a stadiometer (Seca 213, Hamburg, Germany) and weight will be measured using scales (Seca 813, Hamburg, Germany). Body mass index (BMI) will be calculated using these height and weight values. Inclusion and exclusion criteria will also be assessed at this point.

#### Executive function measures

Executive function will be measured using the Cambridge Automated Neuropsychological Test Battery (CANTAB), the verbal fluency task from the Delis Kaplan Executive Function System [32], and the Behavior Rating Inventory of Executive Function – Adult Version® (BRIEF-A) [33].

CANTAB® is an automated, computerised, cognitive test battery that is considered to be the gold standard in cognitive testing. The tests are language independent, have parallel forms to allow repeat testing, are well validated, and have a large normative database. Participants will complete six executive function-related tests over a period of approximately 60 minutes. These are: 1) attention-switching (ability to switch attention between the direction of an arrow and its location on the screen and to ignore task-irrelevant information in the face of interfering or distracting events; taking approximately 7 minutes to complete), 2) intra-extra dimensional set shifting (a test of rule acquisition and reversal, similar to the Wisonsin Card-Sorting test; taking approximately 8 minutes to complete), 3) spatial working memory (a test of the participant’s ability to retain spatial information and to manipulate remembered items in working memory; taking approximately 8 minutes to complete), 4) stockings of Cambridge (a spatial planning test; taking approximately 10 minutes to complete), 5) choice reaction time (a 2-choice reaction time test involving two possible stimuli and two possible responses; taking approximately 7 minutes to complete), and 6) the stop-signal test (a measure of an individual’s ability to inhibit a prepotent response; taking approximately 20 minutes to complete). All participants will complete a motor screening task first to familiarise themselves with the touch screen system.

The verbal fluency task (D-KEFS) [32] requires participants to name as many words as they can that begin with each of 3 letters (F, A, and S). They have 60 seconds per letter. Completion of the full task takes approximately 5 minutes. Participants are instructed to avoid names, place names, numbers, repetitions, and the same word with different endings (e.g. fish, fishing, fished). Higher scores indicate high levels of cognitive flexibility. Raw scores are converted to scaled scores normed by age and gender. The verbal fluency task is sensitive to differences between individuals with impaired executive functions and non-impaired controls [34, 35].

Finally, the BRIEF-A [33] is a 75-item standardized paper and pencil measure that captures an adult’s perceived executive function efficiency and self-regulatory ability in their everyday environment. The BRIEF-A takes 8-10 minutes to complete. The self-report version used here taps two global factors of executive function: behavioural regulation (comprised of inhibition, shifting, self-monitoring, and emotional control) and metacognition (comprised of initiation, working memory, planning, organisation of materials, and task-monitoring). The measure includes three additional validity scales measuring Negativity, Inconsistency, and Infrequency. The BRIEF-A is standardized and validated for use with men and women aged 18 to 90 years. The normative sample includes adults from a range of racial/ethnic backgrounds, educational backgrounds, and is matched to U.S. Census data [36]. The BRIEF-A has demonstrated high internal consistency (alphas range from .93-.96), good test-retest correlations (.82-.93 over 4 weeks), and adequate convergent and divergent validity [37].

#### Dietary and physical activity intentions, past behaviour, and automaticity

Dietary intentions will be measured using six items. The stem ‘over the next week…’ will be followed by ‘I intend to avoid eating’ , ‘I want to avoid eating’ , and ‘I expect to avoid eating’ , in relation to ‘non-core snacks like cakes, biscuits, crisps and sweets’ , and ‘I intend to eat’ , ‘I want to eat’ , and ‘I expect to eat’ , in relation to ‘5 portions of fruit and veg a day’.

Past dietary behaviour will be assessed with 2 items: ‘Over the last week…’ ‘I avoided eating non-core snacks like cakes, biscuits, crisps and sweets’, and ‘I ate 5 portions of fruit and veg a day’. Responses to each of these items will be made using a 5-point Likert scale from Strongly Agree – Strongly Disagree.

Dietary automaticity will be assessed using the 4 item Self Report Behavioural Automaticity Index [38] for ‘Eating non-core snacks like cakes, biscuits, crisps and sweets is something…’ and for ‘Eating 5 portions of fruit and veg a day is something…’ , followed by ‘I do automatically’ , ‘I do without having to consciously remember’ , ‘I do without thinking’ , and ‘I start doing before I realise I’m doing it’. Responses ranged from 1 (strongly agree) to 7 (strongly disagree).

The previously described items were altered to measure physical activity intentions, past behaviour, and automaticity, in relation to the target behaviours of ‘doing at least 30 minutes of moderate activity on most days of the week’ and ‘spending less than 2 hours of my leisure time each day sitting down (e.g. watching TV, at the computer, etc)’. Completion of the diet and physical activity intention/past behaviour/automaticity items takes 3-4 minutes.

#### General intelligence

Raven’s Standard Progressive Matrices (SPM) [39] will be used to assess general intelligence. SPM consists of 60 non-verbal multiple choice puzzles where participants must correctly choose from a selection of elements the one that best completes a pictured pattern. The test is well validated and normed for use with individuals 17 years and older [40]. Completion takes 20-30 minutes.

#### Factors influencing reporting style

The 20-item Positive and Negative Affect Schedule (PANAS) [41] will be used to measure general affect, so that the effect of negative mood on reporting can be controlled for. Participants rate the extent to which they have experienced each of ten positive emotions (interested, excited, strong, enthusiastic, proud, alert, inspired, determined, attentive, active) and 10 negative emotions (afraid, distressed, upset, guilty, scared, hostile, irritable, ashamed, nervous, jittery) over the last few weeks, using a five-point scale from ‘very slightly or not at all’ to ‘very much’. There is good reliability and validity evidence for the PANAS [42] and the measure takes approximately 3 minutes to complete.

The 16-item Social Desirability Scale [43] is a measure of the tendency to present oneself in a socially desirable way. Participants will be asked whether a range of socially desirable but infrequent and socially undesirable but frequent behaviours are things that they do (‘true’ or ‘false’). Completion takes around 5 minutes. Higher scores indicate greater social desirability bias. This measure is used to control for the effects of social desirability bias in questionnaire reporting and has adequate reliability and validity [43].

#### Subjective social status

A visual scale represented by a ten-rung ladder will be used to measure individual’s subjective social status. Participants are instructed to place an ‘x’ on the rung that they feel best represents where they stand at their current point in life, relative to other people in the UK, in terms of wealth, education, and job status. Completion takes less than a minute. Subjective social status has been shown to be a good predictor of health status and health status change [44].

Test order will be randomly determined for each participant using a random number generator. At the end of the laboratory session participants will be given a booklet containing information relevant to the week of real-time assessment. This will contain a study overview, researcher contact details, descriptions of each of the measurement devices to be used, and some frequently asked questions. Additionally, the booklet will contain descriptions of the non-core snack foods of interest in the study, descriptions of sedentary, moderate, and vigorous activity, and definitions of fruit and vegetable portion sizes. The research team member will talk through the information book with the participant and answer any questions regarding the real-time measurement phase of the study.

### Real-time measurement phase

Real-time measurements of snacking, fruit and vegetable consumption, physical activity, mood, context, and executive function will be recorded using the PRO-Diary (CamNtech Ltd., Cambridge, UK). The PRO-Diary is a compact wrist-worn electronic diary that will be used to gather self-reported data. The device measures 5.1 × 3.4 × 0.8 cm and weighs 16 g. The rechargeable battery lasts for up to 30 days (depending on usage). The PRO-Diary is wrist-worn so participants are unlikely to leave it somewhere, as they might with a similar non-wrist-worn device e.g. a PDA. Additionally, the device records the time of an event and therefore data can be time-matched to data from other measures (i.e. GPS and activity monitor). There is little available validity evidence for this device, but responses seem to correspond well with those made using traditional pen and paper scales [45]. The PRO-Diary will be used in the present study to measure the behaviours and psychological constructs of interest; namely non-core snack food consumption, portions of fruit and vegetables consumed, time spent in moderate and sedentary behaviours, mood, context, and executive function.

#### Schedule

The diaries will use a fixed-interval measurement schedule, giving an audible alarm (beep) every 60 minutes between the hours of 7.50am and 9.50pm. This assessment schedule was chosen to (a) minimise recall errors associated with longer assessment windows, (b) ensure that all episodes of snacking over the day are captured, (c) to minimise interruption to scheduled tasks likely to begin on the hour (e.g. appointments, classes, meetings), and (d) to allow data to be subdivided into 1 hour periods to facilitate integration with data from multiple other devices. The diaries include a snooze function to delay inconvenient responses. If the participant chooses to snooze or ignore the diary, it will beep again in 20 minutes (i.e. 10 minutes past the hour).

#### Food intake

The target dietary behaviours to be measured are eating ‘non-core’ snack foods and eating fruits and vegetables. Participants will be presented with the statement ‘Over the last hour’, and will respond ‘yes’ or ‘no’ , in relation to having eaten any of the following snack foods: Chocolate/sweets, Biscuits/cakes/pastries, Crisps/savoury snacks, Savoury pies/pastries, Takeaway /fast food, Soft drinks, and Alcohol. These snacks are defined as non-core foods; that is foods that are surfeit to daily requirements, and are not classified within any of the main food groups recommended for daily consumption [46]. If participants respond ‘yes’ to having eaten any of these snacks, they will then be asked to indicate the portion size from the following options: small, typical, or large/multiple. In order to assess fruit and vegetable consumption participants will report how many portions they have consumed in the past hour, on a scale of 0 to 5.

#### Self-reported activity/sedentary behaviour

The target activity behaviours are time spent in moderate/vigorous physical activity and time spent in sedentary behaviours. Participants will be presented with the statements ‘Minutes moderately/vigorously active?’ and ‘Minutes sitting?’ , and will respond on a scale of 0 to 60 minutes (in 5 minute increments) for each. As participants’ interpretations of terms such as active and sedentary may vary markedly, they will be provided with definitions and examples of each construct in the participant information booklet. Again, these questions will be asked in relation to ‘over the last hour’.

#### Mood

Information on mood will also be collected. We are specifically interested in three aspects of affect. These are hedonic tone, tense arousal, and energetic arousal [47]. These three dimensions are important for the current study research questions because positive affect (hedonic tone) is known to replenish self regulatory resources [48], and stress (tense arousal), perceived tiredness, and lack of energy (energetic arousal) are all associated with self-regulatory depletion [49, 50]. Each of the sub-components of affect will be represented by two items. These are happy and sad (hedonic tone), calm and stressed (tense arousal), and alert and tired (energetic arousal). These items will be rated on uni-polar scales from 0-100, anchored with ‘Not at all’ and ‘Extremely’. Responses for each item will be in relation to ‘Just before the beep, I was…’.

#### Contextual factors

Information on context will be recorded because we are interested in the association between individual’s decisions in relation to their social and physical environment. Using the prompt ‘Just before the beep, I was…’, participants will provide information on 1) their level of hunger (response on a sliding scale anchored with ‘Not at All’ and ‘Extremely’), 2) who they are with (response options include alone, friends, family, colleagues, other), 3) where they are (response options include home, work, outdoors, car, shops/pub/restaurant, other), and 4) what they are doing (response options include work, domestic chores, childcare, socialising, travelling, TV/computer, sports/exercise, eating/drinking, nothing, other).

#### Executive function

Finally, immediately following response to these questions participants will complete a specially designed objective assessment of their executive function levels, known as a GoNoGo task. This task requires the rapid initiation and inhibition of responses, providing a measure of a participant’s ability to regulate behaviour in line with demands. Participants are presented with a randomly ordered sequence of letters (Ms and Ws) each displayed for 500 ms. Inter stimulus interval (ISI) varies from trial to trial between 5 different (but equi-probable) intervals: 1300 ms, 1500 ms, 1700 ms, 1900 ms, and 2100 ms. Participants are asked to respond as quickly as possible to Ms (‘Go’) by pressing a button on the diary, and to make no response to Ws (‘NoGo’). Participants will complete a block of 50 experimental trials every hour, comprising 40 Ms and 10Ws (80%:20% ratio of Go:NoGo). Reaction time to Go stimuli and incorrect responses to NoGo stimuli are recorded. Greater numbers of commission errors (i.e. Go to NoGo stimuli) indicates poorer inhibition. As stimulus order is not predictable, practice effects on repeat assessment of this task are likely to be minimal. Participants will spend approximately 3 minutes every hour completing the questions and GoNoGo task. This will be shorter if the participant indicates that they have consumed no food during the preceding hour, as the question schedule is designed to skip the food-related questions in such cases.

#### Objectively measured physical activity

In addition to the self-reported diary entries, physical activity will be continuously objectively recorded using the Actigraph GT3X + accelerometer. The ActiGraph GT3X + is a small (4.6 cm × 3.3 cm × 1.5 cm) and lightweight (19 g) tri-axial activity monitor that provides data on physical activity including activity counts, energy expenditure, steps, and activity intensity (METs). The device will be hip-worn on an elastic belt and activity will be measured across three planes (vertical, front-to-back, and side-to-side). The GT3X + has an inclinometer to determine subject position (e.g. sitting, lying, and standing) and to identify periods when the device is removed. Data are recorded in a raw format, using a sampling rate of 30 Hertz. Data filtering and epoch selection will be performed after data collection; an epoch of 1 minute will be selected to allow for data integration with data from other devices. The device is capable of collecting data for up to 40 days, using its 512 MB of available memory, comfortably accommodating our 7-day protocol. The GT3X + has been shown to be a valid measure of physical activity when worn on the waist [51].

#### Heart rate and heart rate variability

The Actiheart (CamNtech Ltd. Cambridge, UK) is a chest-worn device (attached via ECG pads) that very accurately measures heart rate and physical activity (activity is measured using an accelerometer- piezoelectric element). Available epochs (sampling periods) are 15 s, 30 s, or 1 min, and data can be collected for a maximum of 4 days when set to record heart rate variability data (an indication of the variation in time intervals between heart beats). There is strong validity and reliability evidence for the Actiheart [52]. Using this device average beats per minute (BPM), and the average of the absolute values of successive differences in R-R intervals in msec (mean of successive differences) will be recorded. The latter is highly correlated with vagal sources of heart rate variability (HRV) [53]. Tonic heart rate variability (HRV) may be a physiological indicator of the current level of cognitive, emotional, and behaviour regulation [54, 55]. HRV reflects the effects of sympathetic and parasympathetic neural activity on the beat to beat variation in heart rate. Importantly, the neural structures involved in modulating HRV are also linked to the brain structures involved in the control of higher order goal directed action [56], and higher HRV is linked to several facets of executive functioning, including cognitive flexibility, less negative affect during stress emotion regulation, less experience of negative affect during stress, effective impulse control, and critically for the present study – resisting dietary temptation [57–61]. Participants will be instructed to wear the Actiheart during waking hours only, for the first four days of the 7-day measurement period as the device can only record data for this period of time.

#### Location

Participants’ location will be tracked using the iTrail (BrickHouse Security) GPS device. The iTrail is a small (4.6 cm × 4.1 cm × 1.3 cm) and lightweight (37g) GPS tracking device. Data (map co-ordinates) are collected at 1-minute intervals, and stored across the first 5 of the 7 measurement days (the device only has sufficient battery life to record for 5 days). The iTrail will be positioned on the elastic belt alongside the Actigraph GT3X+. The device has only one button on the front which will be disabled during initialization, meaning that participants will not be able to tamper with the device or press the button unintentionally. Data from this device will be integrated with information from a Geographic Information System (GIS) to provide information about distance from food outlets, distance from home/work, distance from green-space, and distance from recreational facilities. Additionally, because all the data collected using the devices mentioned above are time-stamped; they can be time-matched to investigate how the different behaviours of interest co-occur.

All devices involved in the study will be worn during waking hours only. At the end of the real time measurement period, a member of the research team will collect all measurement equipment from each participant.

### Data processing

The multiple measurements obtained at several time points each day across seven days generates large quantities of data. For each participant a purpose-written computer macro will be used to match data from each device based on date and time stamps. This will be done using the R programming language (R version 3.0.2, R Foundation for Statistical Computing, Vienna, Austria). This will produce a long-form data file where each row corresponds to minute-level data. Any data generated at a higher resolution than 1-sample per minute will be aggregated to the minute-level (i.e. Actiheart energy expenditure data which was recorded using a 15-second epoch). PRO-Diary question data will be replicated across the preceding hour to which the responses corresponded, because these questions relate to ‘over the last hour’. PRO-Diary GoNoGo task data will be replicated over the following hour, as these data will be used to predict subsequent eating and activity behaviour.

### Data analyses and sample size calculations

**Figure 1:**
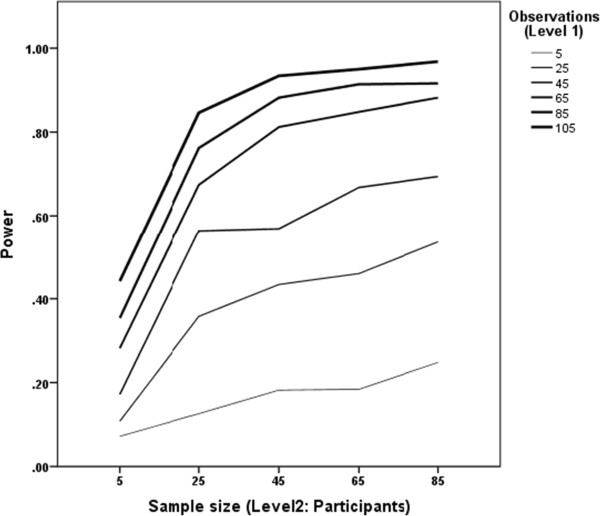
**Multilevel power calculations by sample size and number of observations.**

Statistical analyses will be conducted using SPSS (IBM SPSS statistics version 21, Armonk, NY) and MLwiN (MLwiN version 2.28, Bristol Centre for Multilevel Modelling, UK). Descriptive statistics will be calculated for relevant variables. We will investigate our primary research question using 3-level multilevel models nesting hourly epochs within days within individuals, where significant outcome variability at each level permits [62, 63]. We will build models to test within-person real-time associations of executive functioning (GoNoGo reaction time for correct responses) with snacking behaviour (high calorie intake) and sedentary behaviour (physical activity counts), and cross-level interaction effects of trait (BRIEF-A/CANTAB outcomes) and state (Go NoGo) executive functioning on real-time snacking and sedentary behaviour. Multilevel models will control for time of day, and all epoch-level or day-level predictors will be person-centred. It is very likely in a demanding protocol of this nature that there will be higher than average levels of missing data. However, multilevel modelling is robust to the biasing effects of missing data when maximum likelihood estimation methods are used [64] so imputation of missing data will not be necessary.MLPowSim (Bristol Centre for Multilevel Modelling, UK) and R (R version 3.0.2, R Foundation for Statistical Computing, Vienna, Austria) packages were used for sample size calculations. For the primary research question concerned with the association between executive function at a given time point and subsequent snacking the power calculations estimate a required sample of around 50 participants each with between 65 and 85 observations (i.e. repeated within-person assessments) for a power of 80%. The power curves for a combination of level2 (participants) and level 1 (observations) values are displayed in Figure 1.

## Discussion

The executive functions likely play an important role in controlling goal-directed health behaviours, including snack food consumption. Previous studies have demonstrated the association between executive function and the consumption of unhealthy foods. Specifically, these studies have shown that individuals with weak executive control eat fewer fruits and vegetables and more snacks than intended [10], consume more chocolate given the opportunity to eat freely [12], and that stronger executive control resources are associated with lower frequency of fatty food consumption [8]. Additionally, manipulation of self-control resource via resource-depleting tasks results in reduced capacity to regulate intake of unhealthy foods [15, 16]. However, these studies have only determined cross-sectional between-person associations or obtained findings from contrived laboratory experiments designed to manipulate self-control resource, and little is known about how the executive functions co-vary with behaviours such as snacking within people over time. The SNAPSHOT study aims to determine how within-person fluctuations in the executive functions relate to the deleterious health behaviours of snacking and sedentary behaviour. This study is unique in that it uses momentary assessment techniques to provide ecologically valid data on real-time fluctuations in the efficiency of self-regulatory control.

Gaining a better understanding of how the executive functions co-vary with behaviours such as snacking and sedentary behaviour will provide valuable information about how best to intervene to change such behaviours and will allow theories about the cognitive control of behaviour to be tested within people over time.

## Authors’ information

David McMinn, PhD, Rowett Institute of Nutrition and Health, University of Aberdeen, Health Sciences Building, Foresterhill Campus, Ashgrove Road, Aberdeen, AB25 2ZD. Julia Allan, PhD, School of Medicine and Dentistry, University of Aberdeen, Health Sciences Building, Foresterhill Campus, Ashgrove Road, Aberdeen, AB25 2ZD.

## Abbreviations

BMI: Body Mass Index
BRIEF-A: Behavior Rating Inventory of Executive Function®–Adult Version
CANTAB: Cambridge Automated Neuropsychological Test Battery
DEBQ: Dutch Eating Behaviour Questionnaire
EMA: Ecological Momentary Assessment
GIS: Geographic Information Systems
GPS: Global Positioning Systems
HRV: Heart Rate Variability
IPAQ: International Physical Activity Questionnaire
PANAS: Positive and Negative Affect Schedule
PDA: Personal Digital Assistant
SCG-FFQ: Scottish Collaborative Group Food Frequency Questionnaire
SDS-17: Social Desirability Scale
SPM: Raven’s Standard Progressive Matrices.
